# Four long noncoding RNAs act as biomarkers in lung adenocarcinoma

**DOI:** 10.1515/med-2021-0276

**Published:** 2021-04-21

**Authors:** Zhihui Zhang, Liu Yang, Yujiang Li, Yunfei Wu, Xiang Li, Xu Wu

**Affiliations:** Department of Thoracic and Cardiovascular Surgery/Huiqiao Medical Center, Nanfang Hospital, Southern Medical University, Jingxi Street, Guangzhou, Guangdong 510515, China; Department of Radiology, Charité – Universitätsmedizin Berlin, Berlin, Germany; Department of Emergency Surgery, Nanfang Hospital, Southern Medical University, Guangzhou, Guangdong, China

**Keywords:** lung adenocarcinoma, lncRNA, prognosis, bioinformatics, biomarker

## Abstract

**Introduction:**

Lung adenocarcinoma (LUAD) is currently one of the most common malignant tumors worldwide. However, there is a lack of long noncoding RNA (lncRNA)-based effective markers for predicting the prognosis of LUAD patients. We identified four lncRNAs that can effectively predict the prognosis of LUAD patients.

**Methods:**

We used data gene expression profile for 446 patients from The Cancer Genome Atlas database. The patients were randomly divided into a training set and a test set. Significant lncRNAs were identified by univariate regression. Then, multivariate regression was used to identify lncRNAs significantly associated with the survival rate. We constructed four-lncRNA risk formulas for LUAD patients and divided patients into high-risk and low-risk groups. Identified lncRNAs subsequently verified in the test set, and the clinical independence of the lncRNA model was evaluated by stratified analysis. Then mutated genes were identified in the high-risk and low-risk groups. Enrichment analysis was used to determine the relationships between lncRNAs and co-expressed genes. Finally, the accuracy of the model was verified using external database.

**Results:**

A four-lncRNA signature (AC018629.1, AC122134.1, AC119424.1, and AL138789.1) has been verified in the training and test sets to be significantly associated with the overall survival of LUAD patients.

**Conclusions:**

The present study demonstrated that identified four-lncRNA signature can be used as an independent prognostic biomarker for the prediction of survival of LUAD patients.

## 1 Introduction

Lung cancer is one of the most common cancers worldwide [1]. Adenocarcinoma accounts for 40% of all lung cancers [2]. Worsening of environmental pollution is associated with an increase in the number of patients with the disease. Many new methods are currently used to diagnose and treat this disease [3]. Epidermal growth factor receptor (EGFR) and anaplastic lymphoma kinase are two key oncogenes that trigger lung adenocarcinoma (LUAD) [4,5,6]. Despite great achievements, overall survival (OS) time for LUAD is less than 5 years because of aggressive properties of the tumor and a lack of effective early diagnostic and prognostic biomarkers [7]. Currently, there are only a few prognostic markers for LUAD, and thus identification of valuable prognostic indicators for clinical treatment is very important.

Long noncoding RNAs (lncRNAs) are RNAs that are longer than 200 bp [8]. lncRNAs have been recently shown to regulate large-scale gene expression programs; lncRNAs influence gene expression by interactions across multiple chromosomes and at various locations on a given chromosome. The mechanisms by which some lncRNAs target a distal binding site are not completely clear, and hence, spatial organization analysis of the genome may help to determine the function of lncRNAs. Recent studies suggest that lncRNAs may have extensive effects on the regulation of genes related to tumor formation and metastasis than previously thought [9,10,11]. Moreover, many lncRNAs can be used as predictors of prognosis and survival for tumor patients, providing a reliable basis for cancer diagnosis and treatment. LOXL1-AS1 can be used as a biomarker to assess the prognosis of glioma [12]. AK001796 is an independent predictor of poor prognosis in esophageal squamous cell carcinoma [13]. Prognostic lncRNA signatures have been detected in many cancer types [14,15,16,17]; however, the studies of lncRNAs with the prognosis of LUAD patients are in the early stages and this subject requires long-term efforts.

High-throughput sequencing data and basic clinical information used in the present study were provided by The Cancer Genome Atlas (TCGA). We used high-throughput data and clinical information of 466 LUAD patients in the TCGA database to identify potential lncRNA markers that can effectively predict the survival of LUAD patients. Eventually, we identified four lncRNAs, which were shown to be independent of clinical factors according to stratified analysis, and these four RNAs have not been reported previously. The findings of the present study demonstrated the important role of these four lncRNAs in predicting the survival of LUAD patients.

## 2 Materials and methods

### 2.1 Basic information of the patients

The lncRNA sequencing profile and LUAD clinical information were obtained from the TCGA data set. This study included 466 LUAD patients, excluding the data that lacked complete survival information. Associated clinical information included OS, age, sex, AJCC tumor stage, etc. The patients were randomly divided into two groups, including 233 patients as a training set and 233 patients as a test set. Detailed information on the sets used in the present study is provided in Table 1.

**Table 1:** Clinical characteristics of LUAD patients

Characteristics	Training set (*n* = 233)	Test set (*n* = 233)	Total set (*n* = 466)
**Vital status, *n* (%)**			
Alive	169 (72.5)	178 (76.4)	347 (74.5)
Dead	64 (27.5)	55 (23.6)	119 (25.5)
**Age (years), *n* (%)**			
≤60	81 (34.7)	66 (28.3)	147 (31.5)
>60	152 (65.3)	167 (71.7)	319 (68.5)
**Sex, *n* (%)**			
Female	127 (54.5)	126 (54.1)	253 (54.2)
Male	106 (45.5)	107 (45.9)	213 (45.8)
**Stage, *n* (%)**			
I	124 (53.2)	130 (55.8)	254 (54.5)
II	57 (24.5)	53 (22.7)	110 (23.6)
III	37 (15.9)	40 (17.2)	77 (16.5)
IV	15 (6.4)	10 (4.3)	25 (5.4)
**Mutation status, *n* (%)**			
EGFR	31 (13.3)	28 (12.0)	59 (12.6)
ALK	9 (3.8)	13 (5.5)	21 (4.5)

### 2.2 lncRNA sequencing profile source

LUAD RNA sequencing data were downloaded from the TCGA database (https://tcga-data.nci.nih.gov/tcga/). The data were compared with the human genome, and the reads per thousand bases per million exon models (RPKM) determine the expression levels of lncRNAs and mRNAs. We selected lncRNAs in the TCGA database according to the following three criteria: (1) the transcript was not found in any protein coding regions; (2) the transcribed sequence was found in the GENCODE project [18]; and (3) it was expressed in at least more than half of the LUAD patients. The transcripts with an average RPKM > 0.1 were considered a part of the lncRNA profile of 466 LUAD samples. Finally, 3,881 lncRNAs were identified in the data set. Then, the “edgeR” package for R [19] was used for differential expression analysis based on adjusted *P* < 0.001 and |log_2_(*F*_c_) | ≥ 2 threshold to determine differentially expressed RNAs. Externally validated sequencing data, clinical information, and platform annotation information were obtained from the GSE11969 data set of the Gene Expression Omnibus (GEO) database (http://www.ncbi.nlm.nih.gov/geo). A total of 148 patients were included in this part of the study.

### 2.3 Establishment of the risk formula

Univariate Cox regression was used to calculate the associations between the expression level of each lncRNA and the survival of patients in the training set. lncRNAs with *P* values less than 0.001 were considered to be significant. Then, random forest and multivariate Cox regression models were used to analyze the lncRNAs that had the best independence from the prognosis of the patients. The regression coefficient obtained using multivariate Cox regression model was multiplied by the linear combination of the expression levels of corresponding lncRNAs define the prognostic risk formula. Risk formula = Σ*N*(*i* = 1)(*Expi × Coei*), where *N* represents the total number of prognostic lncRNAs, *Expi* the expression of a certain lncRNA and *Coei* the regression coefficient obtained from the multivariate Cox regression analysis for a certain lncRNA numbered as *i*.

#### 2.3.1 Statistical analysis

Kaplan–Meier survival analysis was used to determine the differences in the OS and progression-free survival (PFS) between the high-risk and low-risk groups. Multivariate Cox regression and stratified analysis were used to determine whether the prognosis prediction based on identified lncRNA is independent of clinical variables. Receiver operating characteristic (ROC) curves were used to compare the sensitivity and specificity of survival prediction based on the risk score. The R package “maftools” was used to analyze mutated genes in LUAD. All analyses were performed using R software (version 3.6.3).

## 3 Results

### 3.1 Differentially expressed lncRNAs

**Figure 1:**
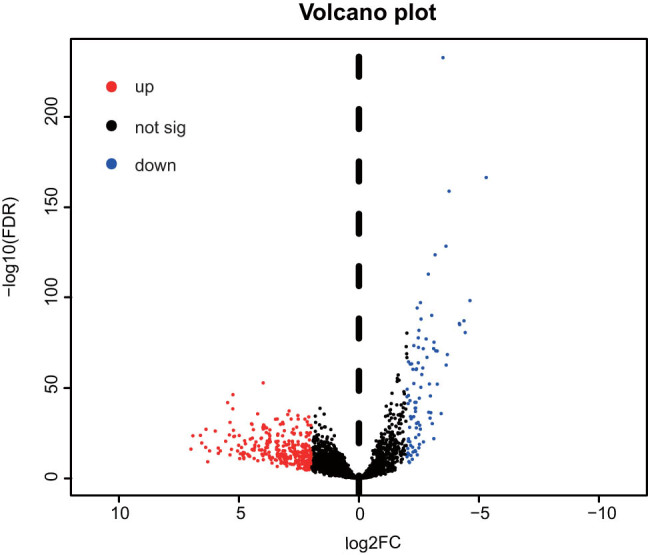
Identification of 381 differentially expressed lncRNAs. Red dots represent 291 upregulated lncRNAs and blue dots represent 90 downregulated lncRNAs, and the intermediate black dots represent lncRNAs without significant differences. lncRNA, long non-coding RNA; FC, fold change; sig, significance; up, upregulated; down, downregulated; FDR, false discovery rate.

A total of 381 differentially expressed lncRNAs were identified according to the criteria of |log_2_(*F*_c_) | ≥ 2 and *P*_adj_ < 0.001. These lncRNAs included 291 upregulated and 90 downregulated lncRNAs (Figure 1). Subsequent prognostic analysis used lncRNAs with expression levels significantly different between the LUAD and control groups.

### 3.2 Identification of four lncRNAs that predicted prognosis in the training set

A total of 466 LUAD patients were randomly divided into a training set (*n* = 223) and a test set (*n* = 223). Univariate Cox regression analysis based on lncRNAs in the training set was used to identify a total of 43 genes (*P* < 0.05) for subsequent analysis. Multivariate Cox regression analysis based on age, sex, and AJCC stage identified four lncRNAs as independent prognostic biomarkers for LUAD patients. These four lncRNAs included AC018629.1, AC122134.1, AC119424.1, and AL138789.1. Three of these lncRNAs (AC018629.1, AC122134.1, and AL138789.1) were positively associated with survival, indicating that higher expression levels of these lncRNA are associated with shorter survival of the patients. The negative coefficient estimated for the remaining lncRNA (AC119424.1) suggested that high expression level was associated with longer survival. Detailed information about these four lncRNAs is presented in Table 2.

**Table 2:** Four lncRNAs significantly associated with the OS

Gene symbol	Ensemble ID	Coefficient	Hazard ratio	95% CI of HR	*P* value
AC018629.1	ENSG00000279806	0.22	1.25	1.10–1.42	5.97 × 10^−4^
AC122134.1	ENSG00000261298	0.11	1.12	1.01–1.24	2.83 × 10^−2^
AC119424.1	ENSG00000243384	−0.22	0.81	0.70–0.93	3.00 × 10^−3^
AL138789.1	ENSG00000233589	0.14	1.16	1.01–1.33	3.97 × 10^−2^

### 3.3 Evaluation of the prognostic model in the training set

**Figure 2:**
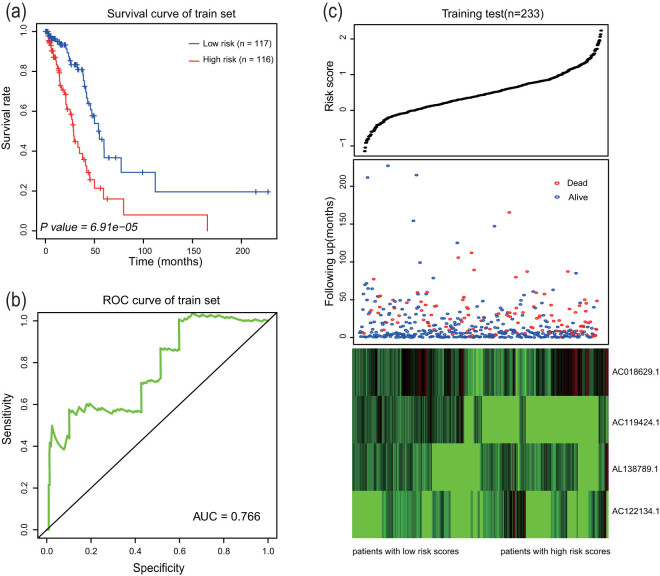
The four-lncRNA associated risk score model predicted the OS of LUAD patients in the training set. (a) Kaplan–Meier assessment of the survival of patients with LUAD in the high-risk and low-risk groups. *P* value represents the difference between the two curves according to the results of two-sided rank test. (b) The ROC curves were used to calculate the AUC and the sensitivity and specificity to evaluate the performance of the score. (c) Distribution of the four-lncRNA based risk score distribution, survival status of the patients, and heatmap expression profiles of four lncRNAs.

The expression levels of these four lncRNAs and their prognostic values obtained by multivariate Cox regression analysis were used to design a risk score formula to predict the survival of patients with LUAD. The risk score formula was as follows: risk score = (0.22 × the expression level of AC018629.1) + (0.11 × the expression level of AC122134.1) + (−0.22 × the expression level of AC119424.1) + (0.14 × the expression level of AL138789.1). The risk score was calculated for each patient in the training set based on the lncRNA signature, then the median risk score was used as a threshold to divide LUAD patients into high-risk (*n* = 117) and low-risk groups (*n* = 116). Additionally, Kaplan–Meier analysis was used to assess the effects of these prognostic indicators on the survival of patients in the training group. The Kaplan–Meier analysis showed that patients in the high-risk group had worse outcome compared with that in the low-risk group (*P* = 6.91 × 10^−5^; Figure 2a). The 3-year OS rate after diagnosis in the low-risk group was 87%, and the 5-year OS rate after diagnosis in the low-risk group was 77%; the 3-year and 5-year survival rates in the high-risk group were only 71 and 67%, respectively. Time-dependent ROC curves were used to evaluate the prognostic significance of four lncRNAs. The area under the curve (AUC) of the four-lncRNA signatures was 0.766, indicating good prognostic value for survival prediction of the patients (Figure 2b). Univariate and multivariable Cox regression analyses showed that risk score based on four lncRNAs was significantly associated with patient survival (Table 3). The basic information about the risk score, survival rate, and expression profiles of four lncRNAs in the samples of the training data set are shown in Figure 2c. The distributions of the risk score, survival rates, and expression profiles of four lncRNAs in the samples of the training set were ranked according to the risk scores. Patients with a high risk score had shorter survival compared with that of patients with a low risk score. In patients with high risk scores, the expression of three lncRNAs (AC018629.1, AC122134.1, and AL138789.1) was significantly upregulated, and the expression of one lncRNA (AC119424.1) was downregulated.

**Table 3:** Univariable and multivariable Cox regression analyses in each data set

Variables	Univariable model^a^			Multivariable model		
	HR	95% CI of HR	*P* value	HR	95% CI of HR	*P* value
**Training set (** ***n*** **= 223)**						
Four-lncRNA risk score	1.551	1.382–1.741	<0.001	1.614	1.410–1.846	<0.001
Gender	0.919	0.561–1.503	0.736	1.132	0.687–1.865	0.627
Age	1.007	0.982–1.032	0.56	1.046	1.019–1.075	<0.001
**AJCC stage**						
(II vs I)	2.593	1.163–4.166	<0.001	3.122	1.549–6.292	<0.001
(III vs I)	3.379	2.120–5.386	<0.001	3.442	1.739–6.811	<0.001
(IV vs I)	3.012	1.524–5.594	<0.001	4.987	2.091–7.139	<0.001
**Testing set (** ***n*** **= 223)**						
Four-lncRNA risk score	1.094	0.971–1.233	0.014	1.112	0.984–1.258	0.009
Gender	1.489	0.855–2.591	0.159	1.799	0.996–3.248	0.051
Age	1.022	0.994–1.502	0.124	1.018	0.989–1.048	0.226
**AJCC stage**						
(II vs I)	0.462	0.157–1.361	0.016	1.85	0.911–3.758	0.086
(III vs I)	0.781	0.251–2.424	0.066	4.913	2.476–9.751	<0.001
(IV vs I)	2.012	0.659–6.413	0.021	2.411	0.785–7.401	0.124
**Entire set (** ***n*** **= 446)**						
Four-lncRNA risk score	1.216	1.135–1.303	<0.001	1.247	1.156–1.345	<0.001
Gender	1.152	0.800–1.657	0.447	1.301	0.890–1.902	0.174
Age	1.014	0.996–1.033	0.13	1.026	1.008–1.045	0.005
**AJCC stage**						
(II vs I)	4.678	2.376–9.211	<0.001	2.844	1.772–4.564	<0.001
(III vs I)	4.434	2.231–8.816	<0.001	4.641	2.888–7.458	<0.001
(IV vs I)	5.15	2.060–9.874	<0.001	3.749	1.873–7.501	<0.001

a In univariable and multivariable Cox regression analyses, risk score, gender, and age were evaluated as continuous variables. AJCC stages were evaluated as category variables. *P* < 0.05 was considered statistically significant in all analyses.

### 3.4 Prognostic value of four-lncRNA signatures and analysis of PFS

**Figure 3:**
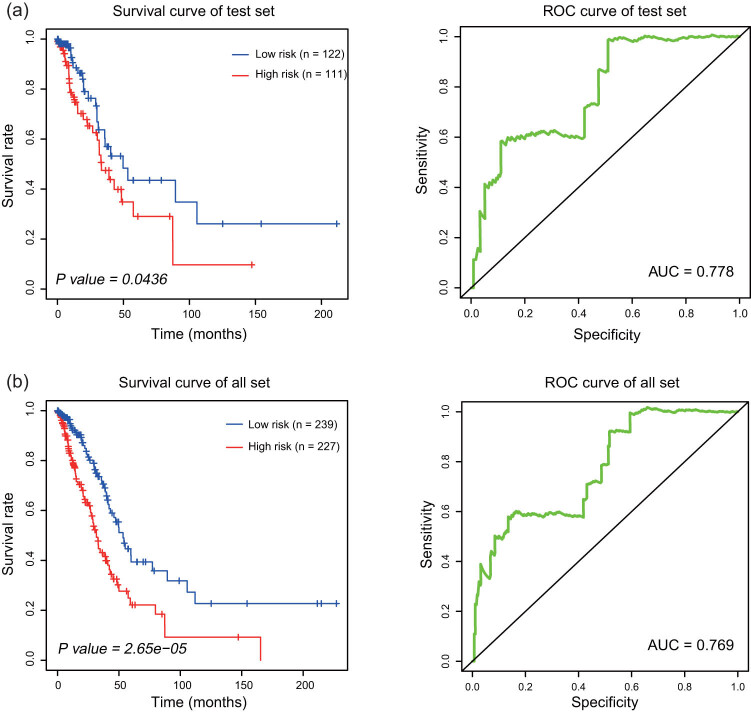
Prognostic value of four-lncRNA signature in the test set and entire set. (a) Kaplan–Meier curve and ROC curve analysis in the test set (*n* = 233). (b) Kaplan–Meier curve and ROC curve analysis in the entire set (*n* = 466).

**Figure 4:**
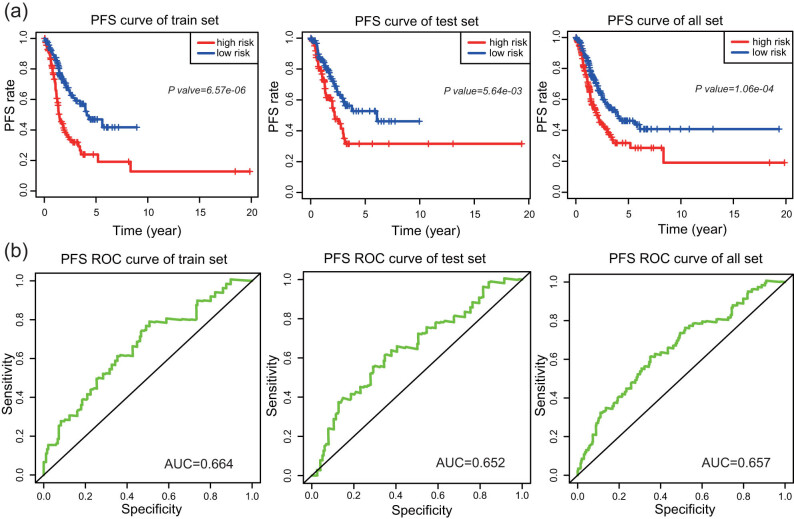
PFS analysis of the four-lncRNA signature. (a) The Kaplan–Meier curve of PFS. (b) The ROC curve of PFS in the training set, test set, and entire set.

The test set and the entire set were used to validate the prognostic model based on four lncRNAs. The risk score for each patient in the test set was calculated according to risk score formula. LUAD patients in the test set were divided into high-risk (*n* = 111) and low-risk groups (*n* = 122) using the threshold similar to that for the division of the training set. Kaplan–Meier analysis was performed in the test set. The results indicated that the survival time of patients in the high-risk group was significantly shorter than that in the low-risk group (*P* = 0.0436). The AUC obtained by ROC curve analysis in the test set was 0.778 (Figure 3a). We analyzed the entire data set in a similar manner. A high risk score based on the four-lncRNA signature was associated with poor OS of LUAD patients (*P* = 2.65 × 10^−5^). The AUC obtained by time-dependent ROC curve analysis was 0.769 (Figure 3b). Then we analyzed the PFS rate in the training set, test set, and the entire set (Figure 4a). The data indicated that the PFS of patients in the high-risk group was significantly lower than that in the low-risk group, and the areas under the ROC curves (Figure 4b) were 0.664, 0.652, and 0.657 in the training set, test set, and the entire set, respectively. The results showed that the predictive value of the signature based on four lncRNAs had great potential for predicting prognosis.

### 3.5 Independence of the four-lncRNA-based biomarkers and clinical factors

**Figure 5:**
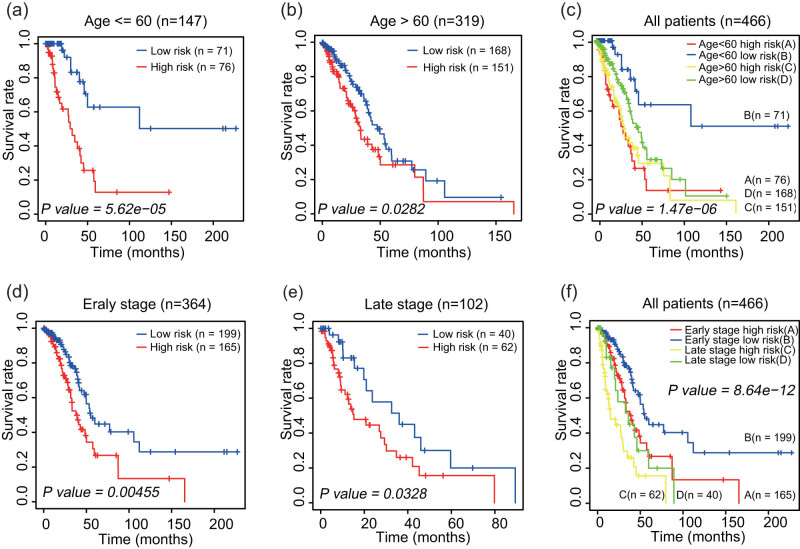
Analysis of associations of four-lncRNA in the groups with various age and tumor stages. (a) The Kaplan–Meier curve of young LUAD patients. (b) Kaplan–Meier curve of elderly LUAD patients. (c) The Kaplan–Meier curve of the entire LUAD patients. (d) The Kaplan–Meier curve of LUAD patients in early stage. (e) Kaplan–Meier curve of LUAD patients in late stage. (f) The Kaplan–Meier curve of the entire LUAD patients. The *P* value represents the difference between the two curves according to two-sided log rank test results.

The predictive ability of the four-lncRNA signature in combination with clinical factors was used to determine whether the signature is independent from other factors associated with survival. These factors included lncRNA risk score, sex, age, and AJCC tumor stages (Table 3). The data showed that the four-lncRNA risk score remained closely associated with the survival rate. Then, entire data set was assessed by stratified analysis. The patients were divided based on the threshold age of 60 years into the younger (*n* = 147) and older groups (*n* = 319) according to a threshold age of 60. LUAD patients in each group were divided according to the risk score into the high-risk and low-risk subgroups. The results suggested that the four-lncRNA signature can predict the prognosis of patients regardless of age (Figure 5a–c). We also analyzed the AJCC staging of the tumors. Stages I and II were classified as early stage (*n* = 364), and stages III and IV were classified as late stage (*n* = 102). The results suggest that the four-lncRNA risk score can adequately predict the survival of patients with LUAD (Figure 5d–f).

### 3.6 Analyses of somatic mutation and function prediction of four prognostic lncRNAs

**Figure 6:**
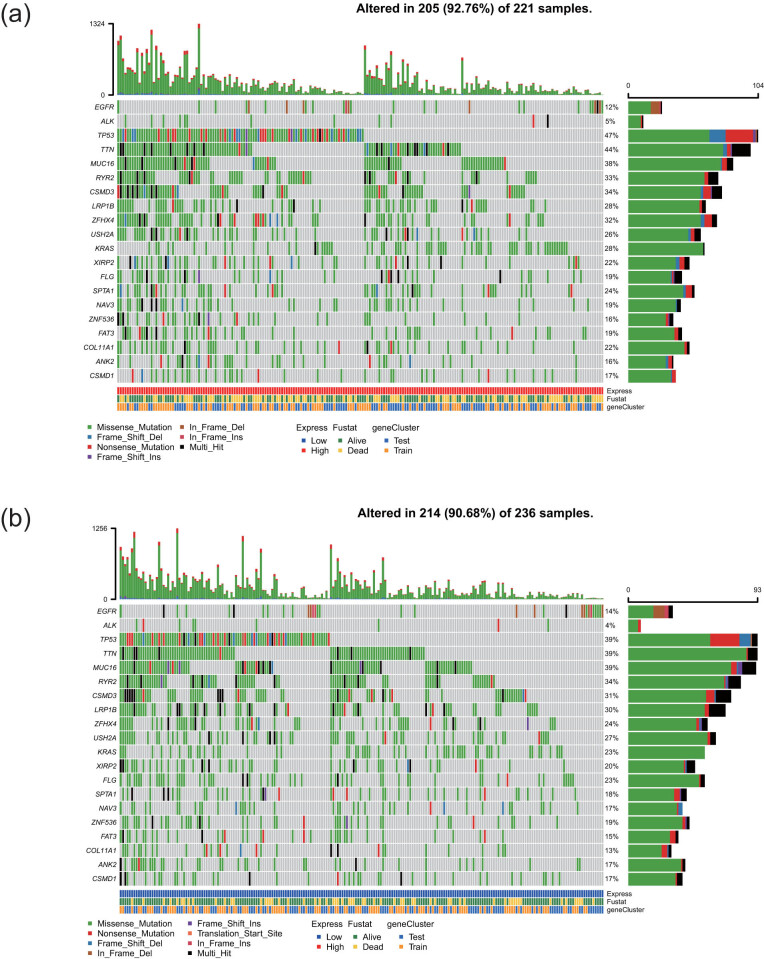
Analysis of somatic mutations in high-risk and low-risk groups. The waterfall plots of the high- (a) and low-risk (b) groups showing the distribution of mutations in the genes with the highest frequency of mutations. The central panel shows the type of mutation in each sample. The upper panel shows the mutation frequency for each sample. The bar chart on the right shows the frequency and type of mutations. The bottom panel is a legend describing the mutation types.

**Figure 7:**
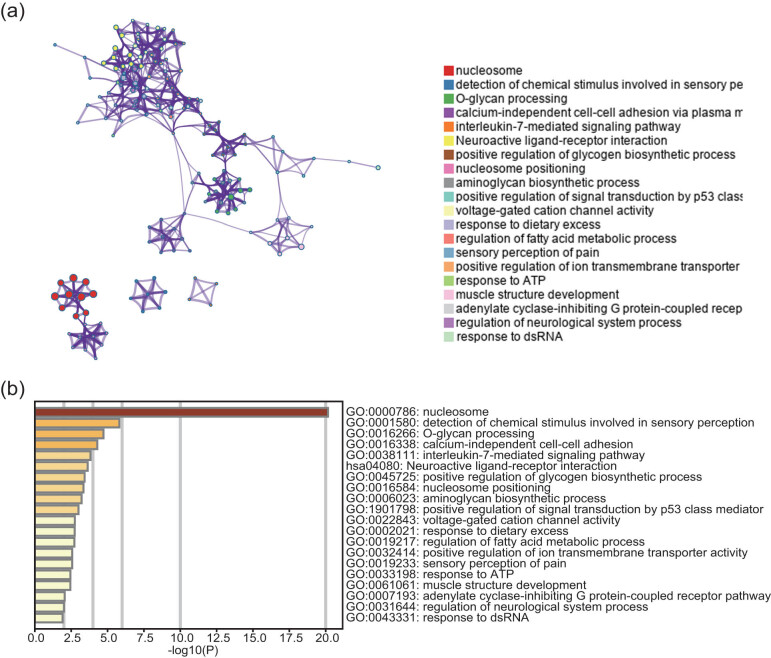
Functional enrichment results of the co-expressed protein-coding genes with four lncRNAs. (a) Significantly enriched pathways of the 232 correlated genes. (b) Functional enrichment diagram of the pathway. Each node represents a GO item. Node size represents the number of genes in the pathway.

Tumor-specific mutations are one of the important factors affecting the prognosis of the patients, and thus we analyzed the gene mutations in the high-risk group and low-risk groups by using a waterfall diagram (Figure 6). TP63 and TTN were the genes with a high mutation frequency. EGFR accounted for 12 and 14% of mutations in the high-risk and low-risk groups, respectively, and ALK accounted for 5 and 4% of the mutations. The functions of four lncRNAs were predicted using coexpression network to investigate the mechanism of action of these lncRNAs in LUAD in detail. Spearman coefficients were calculated based on the expression of lncRNAs and protein-coding genes, and genes with a correlation coefficients >0.4 were selected. A total of 232 genes were used for pathway enrichment analysis. The web-based tool Metascape (http://metascape.org/) was used to analyze the biological function of these coexpressed genes. Most of these genes were enriched in 20 pathways (Figure 7), including interleukin-7-mediated signaling, nucleosome positioning, and regulation of glycogen biosynthetic process. These results suggested that four prognostic lncRNAs may be involved in glycogen metabolism and tumor immunity.

### 3.7 Validation of external databases

**Figure 8:**
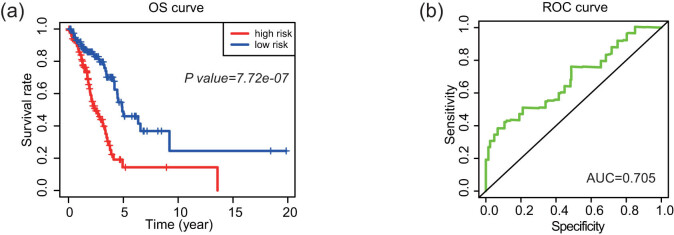
Verification of four-lncRNA signature in the GEO database. Kaplan–Meier survival curves for (a) verifying and ROC curve (b) for calculating the AUCs.

The data of the GEO database were used to validate the four-lncRNA signature. Patients with LUAD in the GSE11969 data set were analyzed. Patients in the high-risk group had lower survival rate than those in the low-risk group, and the AUC was 0.705 (Figure 8). Therefore, four-lncRNA signature had predictive significance for the prognosis of patients with LUAD.

## 4 Discussion

LUAD has become one of the common killers of cancer patients, and its incidence has been recently increasing [20]. The main causes of lung cancer are smoking, air pollution, and gene variation, which have important effects on the development and clinical outcomes of LUAD. Effective therapies have been currently developed to target EGFR, KRAS, and MET; however, the prognosis of patients with advanced lung cancer remains unsatisfactory [21]. The lack of effective and reliable prognostic biomarkers or models is the major problem in enhancing the clinical prognosis of LUAD patients. Recent studies demonstrated that lncRNAs play an important role in the development of tumors. Relevant studies have detected abnormal lncRNA expression in many cancer types, which is closely related to cancer progression [22,23,24]. Therefore, specific lncRNAs can be used as independent diagnostic and prognostic markers in cancer. lncRNAs were rarely reported to predict the prognosis of patients with LUAD, and hence, the present study aimed to provide a more effective prognostic marker for LUAD.

In this study, we analyzed the expression of lncRNAs in LUAD patients and identified four genes as prognostic markers. Subsequent analysis demonstrated that the four-lncRNA signature was an independent predictor of prognosis in patients. The performance of four-lncRNA signatures was evaluated by ROC analysis of the training, test, and entire sets, indicating a good prognostic value of the signature. Additionally, stratified analysis showed that the 4-lncRNA signature was more accurate than other factors in predicting the prognosis of LUAD patients. These four lncRNAs have not been reported previously. Therefore, the enrichment analysis of these lncRNAs was used to understand the details of the regulatory mechanism of lncRNAs.

A large number of references demonstrated that microRNA can be used as diagnostic and prognostic markers [25,26]. For example, abnormally high expression of miR-21-5p is helpful for the diagnosis of non-small cell lung cancer [27]. Technological development resulted in the low costs of microchips and sequencing, and thus, aberrant lncRNA expression in tumors has received increasing attention and has been confirmed to influence the occurrence, development, and metastasis of the tumors. Moreover, lncRNAs have been reported to be more specific biomarkers than microRNAs in some cancer types [21,28,29]. Thus, lncRNAs have shown great potential as new molecular diagnostic biomarkers. For example, upregulation of LINC00963 can inhibit ubiquitination of PGK1 to activate the AKT/mTOR pathway, thus promoting the metastasis of lung cancer [30], and DLX6-AS1 promotes tumor proliferation by regulating the JAK/STAT pathway [31]. The present study demonstrated that multiple lncRNAs predicted the prognosis of LUAD. Kaplan–Meier analysis showed that the risk score model has good prognostic ability. Analysis of the test set and entire set demonstrated the reliability of these biomarkers.

Analysis of the gene mutations in patients with LUAD identified common mutated genes and the genes with the highest mutation frequency. EGFR and ALK are not the most mutated; however, patients with mutations in the EGFR and ALK genes have poor prognosis [32]. These mutations play a very crucial role in tumor surgery, chemotherapy, and precision treatments [33,34,35]. The functions of four lncRNAs identified in the present study are not completely clear; hence, we used enrichment analysis to search for possible biological functions of these lncRNAs. Most of the enriched functions were concentrated in the processes related to nucleosomes, regulation of glycogen synthesis, and interleukin-7 (IL-7)-mediated signaling. Farman et al. demonstrated that the nucleosome position around the transcriptional start site of a tumor suppressor gene in breast cancer is different from that in normal individuals [36]. High levels of glycogen have been reported in most types of cancer cells. The genes involved in glycogen synthesis enable cancer cells to use glucose for anaerobic glycolysis to provide glycogen required during brief energy deficits [37,38,39,40]. IL-7 regulates tumor proliferation, apoptosis, and tumor lymphangiogenesis [41,42,43,44,45]. Therefore, we hypothesized that these four lncRNAs may play an important role in tumor immune regulation and glycogen metabolism in the tumor microenvironment of LUAD.

The present study has several limitations. First, the lncRNA sequencing data were from the TCGA database, and these samples were mainly from African Americans and Caucasians. Therefore, additional studies are needed to determine whether the lncRNA signature is effective in Asian populations. Second, we analyzed and verified four lncRNAs based only on prognostic ability, and hence, the diagnostic and therapeutic capabilities of the signature require more additional investigations. Third, lncRNAs identified in the present study have not been reported in the literature, and further experiments are needed to analyze their functions and mechanisms.

On the whole, based on a large volume of expression profile data, a four-lncRNA signature was shown to have a good prognostic value in LUAD patients. Our results indicate that these four lncRNAs may play an important role in tumor immune regulation and in tumor microenvironment and constitute a reliable biomarker for the prediction of prognosis in LUAD patients.
